# Texture analysis and metabolic parameters of ^18^F-FDG PET/CT to predict primary tumour response and prognosis of paediatric soft tissue sarcomas

**DOI:** 10.1007/s00259-025-07359-z

**Published:** 2025-05-26

**Authors:** Ayşenur Sinem Kartal, Mehmet Oğuz Kartal, Nadide Başak Gülleroğlu, Neriman Sarı, İnci Ergürhan İlhan, Nedim C. M. Gülaldı

**Affiliations:** 1 University of Health Sciences, Department of Nuclear Medicine, Ankara Bilkent City Hospital, Ankara, Türkiye; 2 University of Health Sciences, Department of Pediatric Radiology, Ankara Bilkent City Hospital, Ankara, Türkiye; 3 University of Health Sciences, Department of Pediatric Hematology-Oncology, Ankara Bilkent City Hospital, Ankara, Türkiye

**Keywords:** Soft tissue sarcoma, Paediatric oncology, F-18 FDG PET/CT, Texture analysis, Predictive value

## Abstract

**Introduction:**

We aimed to investigate the value of primary tumour F-18 fluorodeoxyglucose (^18^F-FDG) parameters and textural features in predicting tumour response to neoadjuvant chemoradiotherapy (neo-CRT) and prognosis in paediatric patients with soft tissue sarcoma (STS).

**Materials and methods:**

Twenty-eight paediatric patients with STS who underwent ^18^F-FDG PET/CT studies before neo-CRT were included in this retrospective and single-center study. SUVmax, SUVpeak, SUVmean, metabolic tumour volume (MTV, 40% SUVmax), total lesion glycolysis (TLG), and textural features were extracted from the primary tumour volumes delineated semiautomatically on the baseline PET images. Patients were classified as responders or non-responders according to Response Evaluation Criteria in Solid Tumors 1.1. A receiver operating characteristic (ROC) analysis was performed. The highest AUC values within their respective quantitative groups were selected for further analysis, including logistic regression analysis for response prediction and Cox regression analysis for survival prediction.

**Results:**

In univariate analysis SUVmax > 13.0 (*p* = 0.009), SUVpeak > 12.7 (*p* = 0.017), Histogram Entropy > 0.97 (*p* = 0.036), and NGTDM Busyness < 0.37 (*p* = 0.005) were associated with tumour response for the median follow-up of 25 months. NGTDM Busyness was an independent predictor for the treatment response (OR: 30.5; 95% CI: 1.50-618.5; *p* = 0.026). Age was associated with progression (Cut-off: 11 years, [AUC:0.73 (95% CI: 0,53 − 0,93)] 𝑝=0.022). Progression-free survival outcomes were assessed in aged > 11 years subpopulation. PFS was significantly shorter in patients with high GLSZM_GLNU (*p* = 0,024), GLSZM_ZSNU (*p* = 0,003), and TLG (*p* = 0,016). In multivariate analysis GLSZM_ZSNU > 13,04 (HR: 11.61; 95% CI: 1.35–54.02; *p* = 0.026) was an independent predictor of PFS in subpopulation aged > 11 years.

**Conclusion:**

Heterogeneity texture features Histogram Entropy and NGTDM Busyness and metabolic PET parameters (SUV max and SUVpeak) can predict tumour response. In aged > 11 years patients subgroup analyses, GLSZM ZSNU was an independent factor for PFS.

**Supplementary Information:**

The online version contains supplementary material available at 10.1007/s00259-025-07359-z.

## Introduction

STS, which makes up around 7% of childhood tumours and is the fourth most common malignant tumour group in the paediatric population, is a type of cancer brought on by the unchecked proliferation of numerous mesenchymal cells from muscle tissue [1]. The prevalence of distinct histological subtypes of soft tissue sarcomas (STS) varies across different age groups. Rhabdomyosarcomas are the most common ones and primarily affect children [2].

Current therapeutic paradigms for paediatric sarcomas emphasize a multimodal approach, typically incorporating a combination of neoadjuvant chemotherapy, radical surgical resection, and radiotherapy. Local tumour control in high-grade paediatric soft tissue sarcomas (STS) plays a pivotal role in reducing tumour burden, preventing local recurrence, and enhancing the efficacy of systemic therapy, even in the presence of metastatic disease. A retrospective analysis of the RMS patients demonstrated that aggressive local treatment of the primary tumour significantly improves outcomes, particularly when combined with multimodality therapy [3]. Treatment guidelines recommend a therapeutic approach that emphasizes achieving optimal local control while minimizing the risk of radiation-induced complications and preserving functional outcomes [4].

Several studies have demonstrated the prognostic value of pretreatment ^18^F-FDG PET/CT semi-quantitative parameters may predict outcomes in patients with soft tissue sarcomas [5, 6]. Tumour heterogeneity plays a significant role in treatment resistance and disease progression. Texture analysis, a quantitative imaging approach in radiomics, refers to a set of mathematical methods that quantify the frequency distribution of gray-level intensities of voxels, as well as the spatial relationships between them. Textural analysis offers an effective method for quantifying and characterizing voxel intensity distribution and heterogeneity across diverse imaging modalities. Variations in ^18^F-FDG uptake are commonly observed across the tumour, reflecting underlying factors such as necrosis, cell proliferation, microvessel density, and hypoxia. Numerous studies have demonstrated an association between intratumoural uptake heterogeneity on ^18^F-FDG PET/CT and poor prognosis in several cancers [7]. The prognostic role of PET texture features, the noninvasive assessment of intratumoural heterogeneity, within the primary tumour site in paediatric soft tissue sarcomas remains poorly studied.

In this study, we hypothesize that elevated baseline metabolic parameters and changes in specific textural features are independent predictors of poor treatment response and reduced progression-free survival in pediatric STS reflecting intratumoural heterogeneity. Thereafter, we also expect to prove them if they serve as noninvasive imaging biomarkers.

## Materials and methods

### Patient selection and study design

A retrospective, single-center, cohort study was designed on paediatric patients (< 18 y.o.) and newly diagnosed with STS who underwent ^18^F-FDG PET/CT scans for primary staging before neo-CRT between December 2019 and July 2024. Twenty-eight children were included in the study (Table 1). The inclusion criteria were determined as histopathologically proven disease, and treatment-naive patients with valid baseline ^18^F-FDG PET/CT scan. Patients were included into the study without any tumor location or age preferences to avoid any selection bias. All patients received standardized neoadjuvant chemotherapy protocols based on histological subtype. Patients diagnosed with rhabdomyosarcoma (RMS) were treated according to the Children’s Oncology Group (COG) ARST0431 protocol, which included VAC or interval-compressed VDC/IE regimens for a total of 6 to 9 cycles [8]. Patients with non-RMS soft tissue sarcomas received chemotherapy in accordance with the COG ARST0332 protocol, primarily consisting of ifosfamide and doxorubicin-based regimens, typically for 4 to 6 cycles [9]. Radiotherapy was administered to the primary tumour site in all patients.

**Table 1:** Baseline characteristics of the population

Characteristic	No. of patients
Age (years), median	13 (2–17.7)
Gender, n (%)	
Female	13 (46.4%)
Male	15 (53.6%)
Histologic variant n (%)	
RMS	14 (50%)
Synovial Sarcoma	6 (21.4%)
Extraosseous Ewing Sarcoma	2 (7.2%)
Other	6 (21.4%)
Primary Tumour Site n (%)	
Head and Neck	9 (32.1%)
Abdominopelvic	8 (28.5%)
Genitourinary	3 (10.9%)
Trunk/Extremities	8 (28.5%)
Distant Metastatic at Diagnosis	10 (35.7%)
Treatment Protocol	
COG-ARST0431	14 (%50)
COG-ARST0332	14 (%50)
Post-Treatment MRI Timing (days), median	22 (19–37)
RECIST n (%)	
CR	5 (17.8%)
PR	17 (60.8%)
SD	3 (10.7%)
PD	3 (10.7%)
Relapse/progression	15 (53.5%)
Follow-up (months), median	25 (3–52)
Died	11 (39.2%)

*RMS,* Rhabdomyosarcoma; *COG,* Children Oncology Group; *CR,* Complete response; *PR,* Partial response; *SD,* Stable disease; *PD,* Progressive disease

The exclusion criteria included: (a) prior administration of chemotherapy, radiation, or surgical resection before ^18^F-FDG PET/CT imaging, (b) lack of baseline PET/CT scans, (c) insufficient information regarding therapy, disease stage, and clinical outcomes, (d) lack of pre and post therapeutic MRI scans and (e) presence of secondary malignancy. Only treatment-naive patients were included to ensure a homogeneous therapeutic context. Demographics of the patients (sex, age), histopathological type of tumours, stage, location of the primary tumour, metastasis status at initial staging, baseline ^18^F-FDG PET metabolic parameters, and textural PET features of the primary tumour, treatment response, and survival outcomes were recorded.

### ^18^F-FDG PET/CT imaging protocol

Patients fasted for 4–6 h, and their blood glucose levels were confirmed to be below 120 mg/dL before intravenous injection of ^18^F-FDG (3.7 MBq/kg) for the PET/CT scan. The whole-body PET/CT images were acquired on a GE Discovery IQ PET/CT scanner (GE Healthcare, Milwaukee, Wisconsin, USA) from the vertex to the very distal of the lower extremities. PET acquisition parameters were set at 2.5 min per bed position for each patient. A paediatric imaging protocol was used to enable a smaller field of view, body contour feature (matrix size: 192 × 192, CT helical thickness: 3.75 mm, pitch: 0.938:1, speed: 18.75 mm/rotation, detector rows: 16, beam collimation: 20.0 mm, detector configuration: 16 × 1.25). Tube voltage peak (kVp) and tube current per unit time (mAs) were automatically determined by the scanner based on the children’s body surface area. Images were acquired with the OSEM reconstruction algorithm with GE Healthcare Q-Clear (GE Advance 4.7 PET/CT workstation, 2018).

### Tumour segmentation and textural analysis

**Fig. 1:**
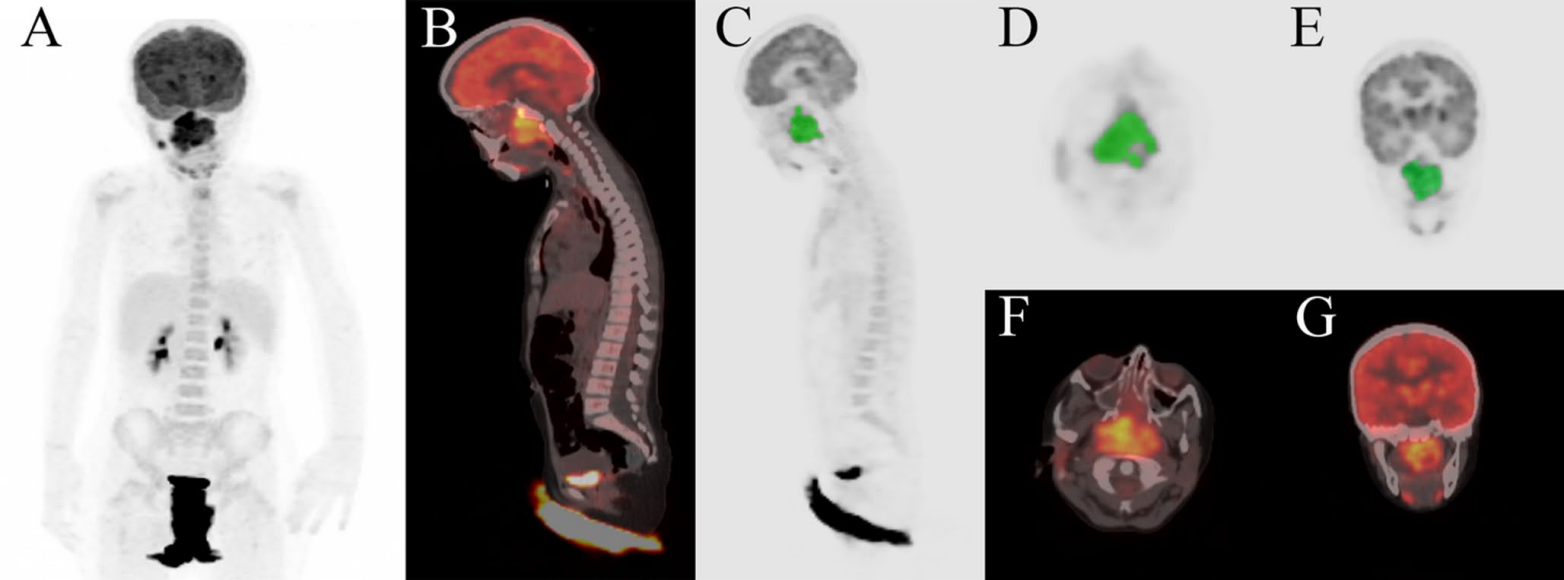
Primary tumour segmentation method based on a 40% SUV threshold in baseline ^18^F-FDG PET/CT image. **A** MIP image, **B** sagittal fusion, primary tumour segmentation in sagittal (**C**), axial (**D**), and coronal (**E**) PET images, axial fusion (**F**), and coronal fusion (**G**) images

The visual assessment included recording the presence of distant metastasis in baseline PET/CT. In the case of distant metastasis, the locations of metastatic lesions (lung, bone, and soft tissue) were documented. For ^18^F-FDG-negative, millimetric pulmonary metastases, chest CT findings were utilized. The quantitative evaluation of the primary tumour included the extraction of maximum, mean, and peak standardized uptake values (SUVmax, SUVmean, SUVpeak) and metabolic tumour volumes (MTV) from pre-treatment PET/CT data using GE Advance 4.7 PET/CT Workstation DICOM data. A 40% SUVmax threshold was utilized to determine MTV. Total lesion glycolysis (TLG) was calculated as the product of metabolic tumour volume (MTV) and mean standardized uptake value (SUVmean). A volume of interest (VOI) was contoured manually over the tumour and a semi-automatic method was used for segmantation based on a threshold of 40% SUVmax. When lesions were located near physiologically high ^18^F-FDG uptake regions (e.g., brain, cervical brown adipose tissue, inflamed tonsils for head and neck; kidney, ureter, bladder for abdominopelvic) or metastatic sites, manual corrections were applied to exclude non-tumoural activity (Fig. 1). Lesion volumes and the distinction from physiological uptake were assessed through consensus delineation by two experienced nuclear medicine physicians. Radiomic features were extracted from PET images using the software LIFEx v.7.4.0 software (Local Image Features Extraction) [10]. Intensity discretization was performed using a bin size of 0.3125 and a total of 64 Gy levels. Voxel intensity was resampled to 64 Gy levels and standardized within absolute resampling limits of 0 to the maximum SUVmax value of the cohort. For each primary lesion, a total of 46 features were extracted from each VOI. The 8 first-order features were: 3 features from shape, 5 from histogram, the 38 s-order textural features were; 6 features from the gray level co-occurrence matrix (GLCM), 16 features from the gray level size zone matrix (GLSZM), 11 features from the gray level run length matrix (GLRLM) and 5 features from the neighboring gray-tone difference matrix (NGTDM).

### Evaluation of treatment response and survival

The treatment response in patients who underwent neo-CRT for primary tumours was evaluated using MRI-based RECIST (Response Evaluation Criteria in Solid Tumours) 1.1 criteria [11], in accordance with current guideline recommendations [12]. Patients who achieved a complete (CR) or partial response (PR) were considered responders (R), while those with stable disease (SD) and progressive disease (PD) were considered non-responders (nR). Patients who underwent surgery without pre or post-neoadjuvant chemoradiotherapy imaging were excluded from the primary lesion response evaluation. Follow-up time was calculated from the start of the therapy date. For patients who experienced disease progression, progression-free survival was defined as the time from therapy to the date of first documented progression. For patients without progression, follow-up time was calculated until the date of the last follow-up imaging. All follow-up times were recorded in months.

### Statistical analysis

The Kolmogorov-Smirnov and Shapiro-Wilk tests were used to assess the normality of data distribution. For normally distributed numerical variables, mean ± standard deviation was reported, while median (minimum, maximum) was reported for non-normally distributed variables. The Mann-Whitney U test was used to evaluate the differences in PET parameters between the R and nR groups. A chi-square test was used to explore the association of the primary tumour response and the metastasis status presence of distant metastasis at initial diagnosis with progression. Receiver operating characteristic (ROC) curves were generated to assess the diagnostic performance of quantitative parameters in predicting primary tumour response and survival. The area under the ROC curve (AUC), sensitivity, and specificity were calculated. Optimal cut-off values for continuous variables were determined using the Youden index (J = Sensitivity(%) + Specificity(%)– 1) derived from receiver operating characteristic (ROC) curves. An univariate logistic regression model was constructed to evaluate the parameter with the highest diagnostic performance in each group, aiming to predict the chemoradiotherapy response of the primary lesion in the entire cohort. A multivariate logistic regression model was constructed using the parameters found to be significant (forward stepwise method). Upon observing that patient age was predictive of progressive disease (optimal cut-off: 11 years; AUC: 0.73), we considered patients older than 11 years as a subgroup warranting further investigation, and therefore subjected them to additional statistical analyses. Progression-free survival outcomes were assessed in a subpopulation aged > 11 years. The impact of groups formed based on the determined cut-off values on progression-free survival was evaluated using the Log-Rank test and Kaplan-Meier method. The Cox proportional hazards model was used to examine the prognostic value of significant parameters on progression-free survival. The Hosmer-Lemeshow test was used to assess the goodness-of-fit of the model. A *p*-value less than 0.05 was considered statistically significant. Statistical analysis was performed using SPSS Statistics version 27 (IBM SPSS Inc., Chicago, IL) and MedCalc Statistical Software version 23.1.5 (MedCalc Software, Mariakerke, Belgium).

## Results

### Cohort characteristics

**Fig. 2:**
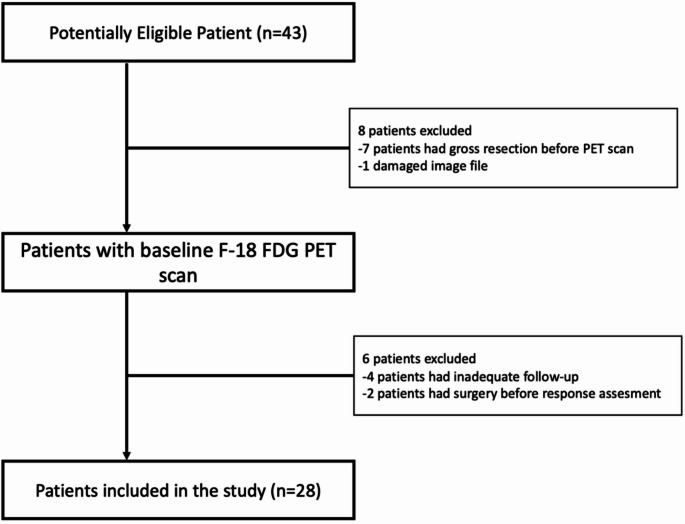
Flow chart diagram summarizing the patient selection process

A total of 28 patients (15 males, median age 13 years, range 2–17.7) met the inclusion criteria. The flowchart of the study is presented in Fig. 2. The histological types included RMS (50%), synovial sarcoma (21.4%), extraosseous Ewing sarcoma (7.2%), undifferentiated sarcoma (7.2%), alveolar soft part sarcoma (7.2%) and malignant peripheral nerve sheath tumour (7.2%). At the time of diagnosis, 35.7% (*n* = 10) of patients presented with metastatic disease. Distant metastases were detected in several organs, such as lung (*n* = 6), liver (*n* = 1), pancreas (*n* = 1), spinal cord (*n* = 1) and non-regional lymph nodes (*n* = 1). In addition, all patients received neoadjuvant chemoradiotherapy as part of their treatment regimens. Complete tumour resection was achieved in 2/28 (7.2%) patients after neoadjuvant chemotherapy. The median follow-up was 25 months (range 3–52). 16/28 (57.1%) patients exhibited disease relapses or progression. During this follow-up period 11/28 (39.2%) patients died from cancer-related causes. Primary tumour response to neoadjuvant therapy was evaluated with post-treatment MR images. Post-treatment MRI scans were performed at a median of 22 days (19–37 days) after the final chemotherapy cycle. Twenty-two of 28 patients were noted as R, while 6/28 were classified as nR. In the R group 5/22 patients had CR, and 17/22 had PR, while in the nR group, 3/6 patients had SD and 3/6 patients had PD. Detailed patient characteristics are summarized in Table 1.

### Prediction of tumour response

22/28 (78.6%) patients were classified as R, and 6/28 (21.4%) patients were classified as nR. Focusing on the relationship with treatment response showed that elevated levels of SUV-based parameters (SUVmin, SUVmean, SUVpeak, and SUVmax) were associated with poor tumour response. No significant association between baseline MTV (*p* = 0.12) and TLG (*p* = 0.65) values and the response to neo-CRT was observed. Several histogram features, such as entropy and uniformity, were associated with tumour response type while other histogram and shape features had no significant association with tumour response type. For the majority of variable levels of baseline textural features showed significant differences between R and nR (Supplemental Table 1). Assessment of the ability to predict tumour response demonstrated that metabolic PET parameters (SUV-based) and several textural features have significant diagnostic performance. Diagnostic performances of metabolic PET parameters and textural features for prediction of tumour response are summarized in (Supplemental Table 2).

In univariate analysis, SUVmax, SUVpeak, Histogram Entropy, and NGTDM Busyness were associated with tumour response (Table 2). In multivariate analysis, a logistic regression model was developed incorporating SUVmax [AUC: 0.84 (95% CI: 0,676-1)], SUVpeak [0.84 (95% CI: 0,676-1)], Histogram Entropy [AUC:0.81 (95% CI: 0,618-1)], GLCM Dissimilarity [AUC:0.87 (95% CI: 0,714-1)], GLRLM RP [AUC:0.87 (95% CI: 0,706-1)], NGTDM Busyness [AUC:0.90 (95% CI: 0,781-1)] and GLSZM LZLGLE [AUC:0.87 (95% CI: 0,706-1)] that demonstrated the highest AUC values within their respective quantitative groups. Appropriate cut-off values were used to categorize variables. In multivariate analysis, baseline NGTDM Busyness < 0.37 (OR: 30.5; 95% CI: 1.50–618.5; *p* = 0.026) was the only independent predictor of tumour response to neoadjuvant chemoradiotherapy (Table 2).

**Table 2:** Univariate and multivariate logistic regression analyses for predictors of treatment response in STS treated by neo-CRT

	Univariate		Multivariate	
	Odds Ratio*	*p* value	Odds Ratio*	*p* value
Age	1.31 (0.97–1.74)	0.075		
Gender	0.67 (0.72–0.98)	0.98		
Metastasic Disease	0.83 (0.13–5.07)	0.84		
SUVmax > 13.0	2.4 (2.14–18.68)	**0.009**	1.43 (0.85–23.08)	0.065
SUVpeak > 12.7	1.2 (1.56–10.23)	**0.017**	5.33 (0.37–7.57)	0.21
Entropy > 0.97	10.71 (1.04–109.7)	**0.036**	3.75 (0.19–7.45)	0.38
GLCM Dissimilarity > 1.44	3.83 (0.94–16.01)	0.061		
GLRLM RP > 0.92	1.83 (0.79–2.68)	0.92		
NGTDM Busyness < 0.37	42.1 (3.03–581.4)	**0.005**	30.5 (1.50–618.5)	**0.026**
GLSZM LZGLE < 0.16	0.92 (0.78–1.08)	0.32		

*Parentheses indicate 95% confidance intervals

**Bold *p* values indicate significant differences

### Prognostic factors for predicting progression-free survival

Median follow-up was 25 (range, 3–52) mo respectively. During follow-up 15/28 (53.5%) patients experienced disease progression or relapse, while 13/28 (46.5%) patients showed no evidence of disease progression. 11/15 patients who had disease progression in follow-up had metastatic disease at initial staging. Pearson Chi-Square test found a statistically significant relationship between progression and metastatic disease at initial staging (𝜒2 = 5.073.32, 𝑑𝑓=1 ve 𝑝=0.024). Disease progression occurred in all patients who showed stable or progressive tumour response (6/6). Fisher’s Exact test found a statistically significant relationship between disease progression and stable or progressive tumour response (𝑝=0.018). Neither PET parameters nor textural features were not associated with disease progression in the entire cohort.

There was a statistically significant age difference in progressive disease (median age 6.59 vs. 14) (*p* = 0.027). Disease progression was significantly more common in the older age group (cut-off: 11 years, [AUC:0.73 (95% CI: 0,53 − 0,93)] 𝑝=0.022). A review of the literature revealed that the adolescent age group in paediatric STS is associated with poor prognosis. Considering the need for further exploration of this population with poor prognosis, a subgroup analysis was conducted for patients aged > 11 years in baseline ^18^F-FDG PET/CT metabolic parameters and textural features. In the aged > 11 years subgroup of 17 patients, disease progression occurred in 14/17 (82.3%). In subpopulation median follow-up and PFS were 14 (range, 3–36) mo and 12 (range, 1–30) mo respectively. The usefulness of the metabolic parameters and textural features measured in the baseline ^18^F-FDG PET/CT to predict progression was established using ROC curves, setting the following values as significant cut-off points. A statistically significant association was observed in SUVpeak, MTV, TLG, GLSZM_GLNU, GLSZM_ZSNU, and NGTDM_Coarseness with progression (Table 3).

**Table 3:** Areas under ROC curves for ability of ^18^F-FDG PET pretreatment parameters to predict progression in aged > 11 years STS patients treated by neo-CRT

	AUC ± SE	95% CI	Sensitivity	Spesificity	Cut-off	𝑝 value
GLSZM GLNU	0,929 ± 0,06	0,794-1	92%	100%	> 4,53	0,023*
GLSZM ZSNU	0,881 ± 0,086	0,713-1	78%	100%	> 13,04	0,044*
NGTDMCoarseness	0,071 ± 0,075	0,782 −1	78%	100%	≤ 0,016	0,023*
SUVpeak	0,881 ± 0,084	0,716-1	85%	100%	> 6,034	0,044*
MTV	0,929 ± 0,075	0,782-1	78%	100%	> 16,4	0,023*
TLG	1,000	1	100%	100%	> 47,81	0,008*

*MTV,* metabolic tumour volume; *TLG,* total lesion glycolysis; *CI,* confidance intervals

**Fig. 3:**
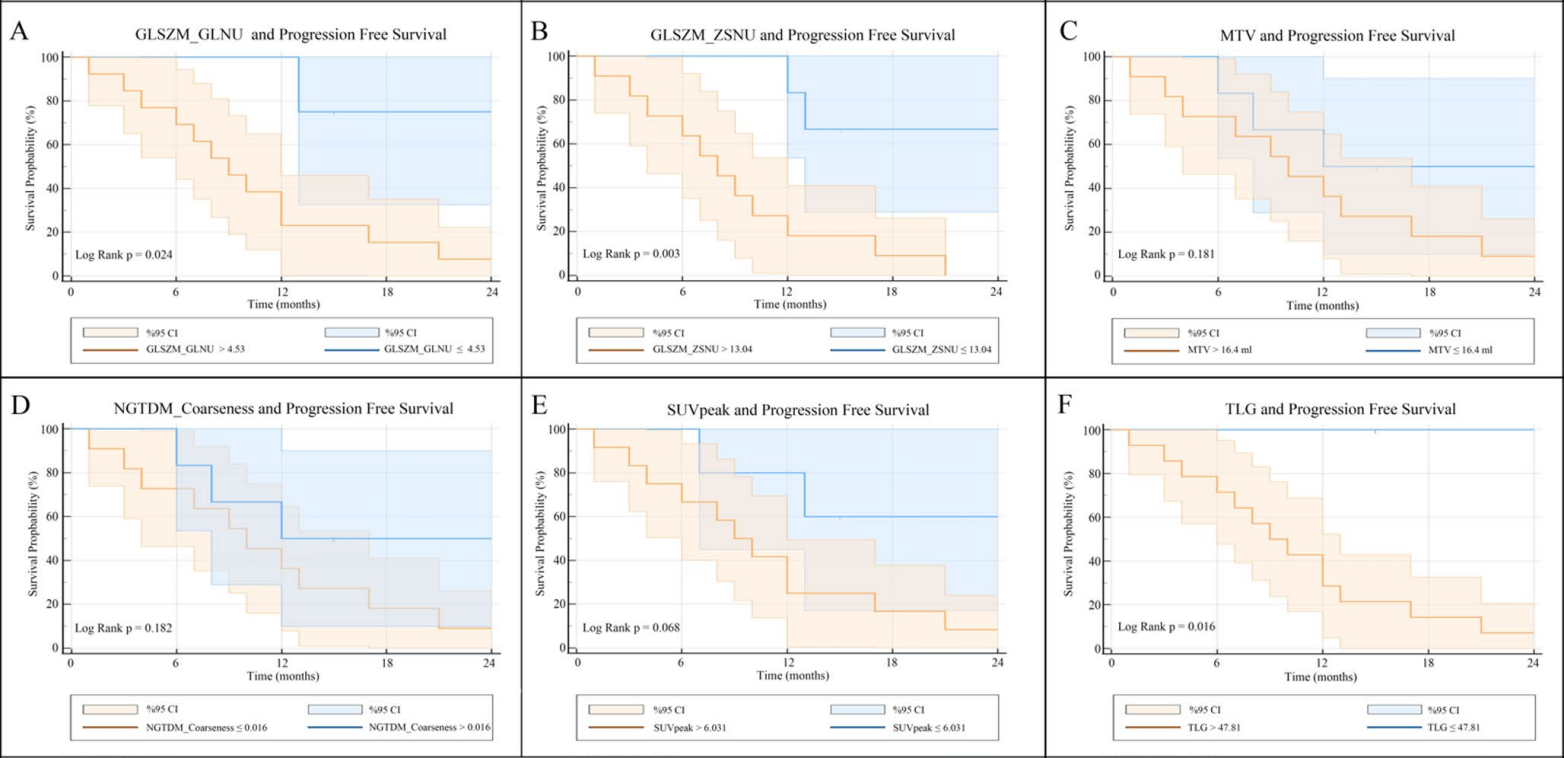
Kaplan-Meier survival curves of the PFS, based on high and low primer tumour GLSZM_GLNU (**A**), GLSZM_ZSNU (**B**), MTV (**C**), NGTDM_Coarseness (**D**), SUVpeak (**E**), and TLG (**F**) values, derived from baseline PET/CT scans in the subpopulation aged > 11 years. The significant differences are highligthed in GLSZM_GLNU (a), GLSZM_GLNU (b) and TLG (f)

In the log-rank analysis, higher values of GLSZM_GLNU (*p* = 0.024), GLSZM_ZSNU (*p* = 0.003) and TLG (*p* = 0.016) were associated with poor PFS (Fig. 3). No statistically significant relationship with PFS was found for the other metabolic parameters and textural features.

Univariate and multivariate analysis showed that GLSZM_ZSNU > 13,04 (HR: 11.61; 95% CI: 1.35–54.02; *p* = 0.026) was and independent factor for PFS in aged > 11 years paediatric patients with STS (Table 4).

**Table 4:** Univariate and multivariate Cox regression analyses for PFS in aged >11 years STS patients treated by neo-CRT

	Univariate		Multivariate	
	Hazard Ratio*	𝑝 value	Hazard Ratio*	𝑝 value
Gender	0.82 (0.45-1.50)	0.53		
Metastasis Status	1.49 (0.45-4.88)	0.54	1.72 (0.49-5.98)	0.39
Response Status	1.72 (0.54-5.41)	0.35	0.95 (0.28-3.27)	0.85
SUVpeak >6.03	3.69 (0.89-3.68)	0.092		
MTV >16,4	2.33 (0.64-8.50)	0.19		
TLG >47,81	3.83 (0.23-6.30)	0.16		
NGTDM Coarseness ≤0,016	2.38 (0.65-8.49)	0.19		
GLSZM ZSNU >13,04	7.32 (1.57-34.02)	**0.011**	11.61(1.35-54.02)	**0.026**
GLSZM GLNU >4,53	3.44 (0.95- 5.85)	0.056		

*MTV,* metabolic tumour volume; *TLG,* total lesion glycolysis

*Parentheses indicate 95% confidance intervals

**Bold *p* values indicate significant differences

## Discussion

The early identification of patients with poor treatment response using baseline imaging biomarkers could potentially support more personalized therapeutic strategies in paediatric soft tissue sarcomas. For instance, high-risk patients identified via ^18^F-FDG PET textural features may benefit from intensified chemotherapeutic regimens, early consideration of radiotherapy, or closer surveillance. This approach may eventually contribute to risk-adapted treatment models. In this study, we demonstrated that different baseline ^18^F-FDG PET image-based metabolic and several textural feature parameters can predict the response to combined chemoradiotherapy. To the best of our knowledge, this has not previously been reported in STS for paediatric age group treated with preoperative neo-CRT.

In this study, baseline PET metabolic parameters of the primary tumour, such as SUVmax and SUVpeak were found to be significant discriminators between R (CR + PR) and nR (SD + PD), whereas MTV and TLG did not show significant predictive value. This finding revealed that higher SUV values, indicative of increased metabolic activity and tumour cell proliferation rates, are linked to more aggressive tumour behavior and worse patient outcomes. As previously proposed for STS [13, 14], all tumour ^18^F-FDG metabolic (SUV) and volumetric measurements (MTV and TLG) seem to be significantly correlated with poorer survival outcomes. Therefore, through their prognostic value as novel imaging biomarkers, ^18^F-FDG PET parameters may play a role in the stratification of high-risk patients and the prediction of post-treatment courses for STS. The high performance of SUVmax in predicting progression has also been reported El-Kholy et al. reported in paediatric RMS which is the most common type in paediatric STS, higher SUVmax values have no significant correlation with EFS and OS [6]. In our study, only SUVpeak in SUV-based parameters, has high diagnostic performance for progression prediction but all SUV-based metabolic parameters were not significantly associated with PFS in subpopulation.

The heterogeneity of tumour ^18^F-FDG uptake is related to many factors including necrosis, cell proliferation, fibrosis, and hypoxia. It has been shown that intratumour heterogeneity can be associated with aggressive tumour behaviour and treatment response diversity [15–17]. Several studies have previously investigated the relationship between ^18^F-FDG PET/CT image analysis and tumour behaviours. Eary et al. improved an image analysis algorithm for quantitation of tumour ^18^F-FDG spatial heterogeneity and demonstrated that highly heterogeneous metabolic uptake distribution is an independent predictor of survival in patients with sarcoma [18]. Textural features on ^18^F-FDG PET/CT, which enables the detection of features and patterns beyond the resolution of human perception, have been progressively expanding in recent years. The significant prognostic value of tumour heterogeneity was assessed through textural features of various tumours shown in numerous studies [19–21]. Although studies on textural analysis in paediatric solid tumours are limited, in a study on paediatric sarcomas, Aydos et al. reported that higher values of NGTDM Contrast and GLSZM ZSNU parameters were associated with poorer survival [22].

NGTDM features such as Busyness exhibited a wide dynamic range across both abnormal and normal tissues, as reflected by their high standard deviations, which capture intensity variations among neighboring voxels. NGTDM Busyness reflects the rate of spatial intensity variation within a lesion and is particularly relevant in the context of PET and CT image texture, as abnormal tissues typically exhibit more rapid intensity changes compared to normal tissues [23]. Yu et al. reported that tumour and positive node had lower busyness than normal tissue [24]. Cook et al. also reported lower busyness, lower coarseness, and higher contrast were able to differentiate subsequent R from nR in non-small cell lung cancer [25]. In our study lower Busyness from NGTDM was an independent predictor for assessing tumour response.

In paediatric STS, patients with metastatic disease have a less than 30% survival rate and are classified as a high-risk group [26, 27]. ^18^F-FDG PET/CT, as a whole-body imaging technique, is effective in identifying distant metastatic sites. In our study population, the presence of metastatic disease at initial staging was associated with poor outcomes.

The study revealed a higher risk of progression among older children in the cohort. This situation has been attributed to various factors, including increasing genomic complexity with age, as well as the delayed onset of symptoms in adolescent patients until the later stages of the disease [28, 29]. A prospective analysis of non-RMS, STS patients under 30 years old reported that those aged 2–10 years had better EFS and OS compared to older patients [9]. The meta-analysis conducted by Yadgarov et al. [30] highlighted a significant impact of age on the association between baseline SUV and survival outcomes in adult STS patients but not in paediatric STS patients. In the adolescent and young adult (AYA) population, defined as aged between 15 and 39 years, a recent study reported that several subtypes of STS, which are chemosensitive in other age groups are chemoresistant [31]. These results indicate that patients in this age group could have unique biological differences compared to older adults and children. Considering the need for further investigation of this population with poor prognosis, a subgroup analysis was performed for patients older than 11 years. The analysis revealed that GLSZM GLNU, GLSZM ZSNU, NGTDM Coarseness, SUVpeak, MTV, and TLG demonstrated high diagnostic performance in predicting progressive disease. GLSZM ZSNU was an independent predictor in multivariate analysis. Several studies analyzing the impact of textural features on survival outcomes have identified ZSNU as a significant prognostic predictor for PFS and OS [32, 33]. GLSZM provides information about how often groups of similar intensity values appear in an image. When the texture is more homogeneous, the matrix becomes wider and flatter. ZSNU quantifies the distribution of the number of zones across different zone sizes within the VOI. A higher ZSNU value indicates that non-uniform distribution of zone sizes, reflecting increased heterogeneity.

MTV has strong predictive power that may come from its ability to show the total metabolic burden of the tumour more completely. This approach, rather than relying on a single point, could result in better predictions of treatment outcomes. A meta-analysis demonstrated that baseline PET/CT volumetric parameters, including MTV have significant prognostic value for overall survival (OS) in patients with STS, providing valuable insights for identifying high-risk patients [34]. In the current study, MTV was not significantly associated with PFS. Despite the lack of statistical significance, MTV indicates a high level of predictive performance. At the optimal cut-off value (> 16,4 cm³) sensitivity was 78% and specificity was 100%, demonstrating the test’s effectiveness in identifying progressive disease (*p* = 0,023).

Several limitations must be acknowledged. The retrospective, single-center nature of the study and the relatively small sample size inherently limit the statistical power. The wide confidence intervals observed in some multivariate regression models, particularly for NGTDM Busyness, likely reflect the limited sample size and the number of observed events. While the statistical significance supports a potential predictive role, the precision of the estimated effect should be interpreted with caution. Our study included various STS subtypes (e.g., rhabdomyosarcoma, synovial sarcoma, extraosseous Ewing sarcoma) and different chemotherapy protocols, reflecting the inherent heterogeneity of this disease group. This heterogeneity may limit the generalizability of certain findings. Although this diversity may limit the generalizability of certain findings, it also reflects real-world clinical complexity and thus may enhance the external validity of our results. Although PET/CT-based response assessment has shown prognostic value in retrospective studies involving paediatric sarcomas, we opted to use RECIST 1.1 criteria based on MRI, which remains the guideline-recommended standard for local response evaluation in paediatric STS. In addition, PET/CT was not uniformly performed post-treatment in our cohort. Finally, the imaging parameters in this study were based on a 40% SUV threshold. While this threshold is commonly used in oncologic PET studies for standardization, it may not fully account for tumour-specific variations in tracer distribution. A standardized threshold for clinical application has yet to be determined.

## Conclusion

In conclusion, primary tumour heterogeneity texture features Histogram Entropy and NGTDM Busyness, as well as metabolic PET parameters (SUV max and SUVpeak) can predict tumour response. All of them have limited value in predicting the prognosis of all paediatric patients with STS. In subpopulation analyses higher tumour heterogeneity, as indicated by textural features, is significantly associated with shorter PFS. Only GLSZM_ZSNU calculated on baseline ^18^F-FDG PET/CT is an independent predictor for PFS in patients aged > 11 years STS patients treated by neo-CRT. Future multicenter studies with larger, more homogeneous cohorts and prospective design are warranted to validate and expand upon our findings.

## Electronic supplementary material

Below is the link to the electronic supplementary material.

Supplementary Material 1

Supplementary Material 2

## Data Availability

The authors declare that all data supporting the findings of this study are available within the article.

## Abbreviations

^18^F-FDG: ^18^F-fluoro-2-deoxy-2-d-glucose
AUC: Area Under Curve
CI: Confidence Interval
CT: Computed Tomography
GLCM: Gray-Level Co-Occurrence Matrix
GLRLM: Gray Level Run Length Matrix
GLSZM: Gray Level Size Zone Matrix
PFS: Progression-Free Survival
HR: Hazard Ratio
MTV: Metabolic Tumour Volume
NGTDM: Neighbouring Gray Tone Difference Matrix
OR: Odds Ratio
PET: Positron Emission Tomography
CRT: Chemoradiotherapy
ROC: Receiver Operating Characteristic
PD: Progressive Disease
STS: Soft Tissue Sarcoma
SUVmax: Maximum Standardized Uptake Value
SUVmean: Mean Standardized Uptake Value
TLG: Total Lesion Glycolysis
