# Supplementary material for: Texture analysis and metabolic parameters of 18F-FDG PET/CT to predict primary tumour response and prognosis of paediatric soft tissue sarcomas
Source: Eur J Nucl Med Mol Imaging. 2025 May 26;52(13):4913–23. doi: 10.1007/s00259-025-07359-z (PMC12589223; doi:10.1007/s00259-025-07359-z)
Supplement: Supplementary file 1 — Supplementary Material 1 [file 259_2025_7359_MOESM1_ESM.docx]

Supplemental Table 1. Comparison of baseline F-18 FDG PET parameters and textural features between responders and non-responders by RECIST in STS treated by neo-CRT

| **Parameters** | **Responder** | **Non-Responder** | **𝑝 value** |
| --- | --- | --- | --- |
| SUVmin | 4,32 (1,08-6,76) | 7,00 (4,57-14,08) | 0,014* |
| SUVmean | 5,7 (1,4-8,82) | 10,09 (6,055-19,02) | 0,029* |
| SUVmax | 9,9 (2,7-16,89) | 17,5 (11,43-35,09) | 0,010* |
| SUVpeak | 8,67 (2,3-15,31) | 17,13 (9,25-30,47) | 0,014* |
| MTV | 92,1 (5,37-761,43) | 28,29 (8,1-261,07) | 0,124 |
| TLG | 496,75 (36,08-4958,85) | 305,75 (52,10-1774,26) | 0,654 |
| Histogram_Skewness | 0,75 (-0,39 – 1,62) | 0,87 (0,28 -1,39 ) | 0,433 |
| Histogram_Kurtosis | 0,13 (-0,09 – 5,30) | 0,14 (-0,73 – 1,68) | 0,911 |
| Histogram_Entropy | 0,82 (0,2-1,08) | 1,14 (0,78-1,36 ) | 0,022* |
| GLCM_Uniformity | 0,18 (0,09-0,76) | 0,087 (0,05-0,21) | 0,029* |
| GLCM_Angular Second Moment | 0,037 (0,009-0,060) | 0,007 (0,002-0,04) | 0,016* |
| GLCM_Contrast | 3,32 (0,23-12,26) | 21,98 (4,04-106,94) | 0,007* |
| GLCM_Dissimilarity | 1,38 (0,21-2,67) | 3,54 (1,47-7,92) | 0,006* |
| GLCM_Inverse Difference | 0,56 (0,40-0,89) | 0,35 (0,23-0,54) | 0,006* |
| GLCM_Correlation | 0,48(0,15-0,63) | 0,41(0,30-0,61) | 0,287 |
| GLRLM_SRE | 0,84 (0,54-0,94) | 0,95 (0,87-0,98) | 0,006* |
| GLRLM_LRE | 1,96 (1,30-11,43) | 1,23 (1,09-1,79) | 0,006* |
| GLRLM_LGLRE | 0,009 (0,0039-0,11) | 0,004 (0,0009-0,0084) | 0,019* |
| GLRLM_HGLRE | 122,5 (10,15-283,45) | 424,8 (130,85-1277,94) | 0,022* |
| GLRLM_SRLGLE | 0,0074 (0,0035-0,061) | 0,0034 (0,0009-0,0075) | 0,019* |
| GLRLM_SRHGLE | 102,25 (5,78-255,87) | 396,58 (117,2-1252,12) | 0,019* |
| GLRLM_LRLGLE | 0,018 (0,006-1,26) | 0,006 (0,001-0,013) | 0,016* |
| GLRLM_LRHGLE | 248,24 (85,66-423,94) | 571,10 (199,27-1386,63) | 0,033* |
| GLRLM_GLNU | 257,51 (13,68-2124,31) | 30,33 (8,68-241,57) | 0,029* |
| GLRLM_RLNU | 1057,41 (93,37-9005,12) | 429,34 (130,32-1498,66) | 0,179 |
| GLRLM_RP | 0,79 (0,4-0,91) | 0,93 (0,82-0,97) | 0,006* |
| NGTDM_Coarseness | 0,0041 (0,0004-0,036) | 0,009 (0,003-0,029) | 0,093 |
| NGTDM_Contrast | 0,061 (0,005-0,22) | 0,24 (0,06-0,81) | 0,014* |
| NGTDM_Busyness | 2,15 (0,20-17,35) | 0,34 (0,61-1,30) | 0,003* |
| NGTDM_Complexity | 60,40 (1,26-272,0) | 513,23 (47,56-3134,38) | 0,012* |
| NGTDM_Strength | 0,27 (0,19-4,28) | 2,13 (0,45-20,06) | 0,009* |
| GLSZM_SZE | 0,52 (0,29-0,64) | 0,57 (0,44-0,76) | 0,179 |
| GLSZM_LZE | 3163,1 (26,8-127970,70) | 16,09 (3,18-1760,07) | 0,007* |
| GLSZM_LGLE | 0,009 (0,004-0,18) | 0,0042 (0,0009-0,009) | 0,033* |
| GLSZM_HGLE | 120,5 (8,8-291,5) | 402,94 (127,2-1292,50) | 0,016* |
| GLSZM_SZLGLE | 0,0047 (0,0023-0,19) | 0,002 (0,0007-0,005) | 0,016* |
| GLSZM_SZHGLE | 57,23 (4,80-168,63) | 237,25 (57,40-971,53) | 0,033* |
| GLSZM_LZLGLE | 32,04 (0,176-9366,9) | 0,083 (0,003-10,14) | 0,006* |
| GLSZM_LZHGLE | 273483 (3478-6585x10^9^) | 6211 (3518-318234) | 0,014* |
| GLSZM_GLNU | 26,01 (3,33-90,58) | 16,99 (4,87-30,84) | 0,287 |
| GLSZM_NGLNU | 0,17 (0,096-0,50) | 0,087 (0,049-0,19) | 0,044* |
| GLSZM_ZSNU | 34,9 (3,66-195) | 55,85 (10,72-142,6) | 0,287 |
| GLSZM_NZSNU | 0,27 (0,01-0,38) | 0,32 (0,21-0,54) | 0,131 |
| GLSZM_ZP | 0,066 (0,013-0,32) | 0,38 (0,96-0,67) | 0,007* |
| GLSZM_GLV | 6,25 (1,0-11,86) | 21,4 (4,75-84,5) | 0,012* |
| GLSZM_ZSV | 2874 (15,81-122242) | 8,3 (0,96-1651,65) | 0,007* |
| GLSZM_ZSE | 5,07 (3,11-6,1) | 5,51 (4,82-5,9) | 0,198 |
|  |  |  |  |
