# Supplementary material for: Texture analysis and metabolic parameters of 18F-FDG PET/CT to predict primary tumour response and prognosis of paediatric soft tissue sarcomas
Source: Eur J Nucl Med Mol Imaging. 2025 May 26;52(13):4913–23. doi: 10.1007/s00259-025-07359-z (PMC12589223; doi:10.1007/s00259-025-07359-z)
Supplement: Supplementary file 2 — Supplementary Material 2 [file 259_2025_7359_MOESM2_ESM.docx]

Supplemental Table 2 Diagnostic performances of baseline F-18 FDG PET parameters and textural features predicting non-responders evaluated by RECIST in STS treated by neo-CRT

| Parameter | AUC±SD | %95 CI | Sensitivity (%) | Specificity (%) | Treshold | 𝑝 value |
| --- | --- | --- | --- | --- | --- | --- |
| SUVmin | 0,83±0,096 | 0,646-1 | 67 | 91 | >5,2 | 0,014 |
| SUVort | 0,79±0,101 | 0,597-0,994 | 50 | 100 | >11,1 | 0,029 |
| SUVmax | 0,84±0,088 | 0,676-1 | 67 | 91 | >13,0 | 0,010 |
| SUVpik | 0,83±0,096 | 0,646-1 | 67 | 86 | >12,7 | 0,014 |
| Histogram_Entropy | 0,81±0,098 | 0,618-1 | 83 | 68 | >0,97 | 0,022 |
| GLCM_Angular Second Moment | 0,82±0,09 | 0,635-1 | 86 | 67 | ≤0,012 | 0,016 |
| GLCM_Contrast | 0,86±0,08 | 0,706-1 | 100 | 64 | >3,86 | 0,007 |
| GLCM_Dissimilarity | 0,87±0,08 | 0,714-1 | 100 | 64 | >1,44 | 0,006 |
| GLCM_Inverse Difference | 0,87±0,08 | 0,706-1 | 100 | 67 | ≤0,4 | 0,006 |
| GLRLM_SRE | 0,87±0,08 | 0,706-1 | 100 | 59 | >0,86 | 0,006 |
| GLRLM_LRE | 0,87±0,08 | 0,706 -1 | 100 | 67 | ≤1,29 | 0,006 |
| GLRLM_LGLRE | 0,81±0,09 | 0,629 -1 | 82 | 67 | ≤0,006 | 0,019 |
| GLRLM_HGLRE | 0,81±0,09 | 0,621-1 | 83 | 64 | >0,83 | 0,022 |
| GLRLM_SRLGLE | 0,81±0,09 | 0,623-1 | 82 | 67 | ≤0,005 | 0,019 |
| GLRLM_SRHGLE | 0,81±0,09 | 0,636-1 | 100 | 55 | >116,3 | 0,019 |
| GLRLM_LRLGLE | 0,82±0,09 | 0,650-1 | 64 | 100 | ≤0,014 | 0,016 |
| GLRLM_LRHGLE | 0,78±0,1 | 0,546-1 | 67 | 96 | >350,1 | 0,033 |
| GLRLM_GLNU | 0,79±0,09 | 0,606-1 | 50 | 100 | ≤276,9 | 0,029 |
| GLRLM_RP | 0,87±0,08 | 0,706-1 | 67 | 100 | >0,92 | 0,006 |
| NGTDM_Contrast | 0,833±0,10 | 0,638-1 | 67 | 96 | >0,19 | 0,014 |
| NGTDM_Busyness | 0,902±0,06 | 0,781-1 | 95 | 67 | ≤0,37 | 0,003 |
| NGTDM_Complexity | 0,841±0,08 | 0,667-1 | 83 | 77 | >135,6 | 0,012 |
| NGTDM_Strength | 0,856±0,08 | 0,698-1 | 100 | 64 | >0,44 | 0,009 |
| GLSZM_LZE | 0,864±0,08 | 0,690-1 | 86 | 67 | ≤62,5 | 0,007 |
| GLSZM_LGLZE | 0,788±0,1 | 0,585-1 | 59 | 83 | ≤0,008 | 0,033 |
| GLSZM_HGLZE | 0,826±0,09 | 0,644-1 | 67 | 82 | >197,4 | 0,016 |
| GLSZM_SZLGLE | 0,826±0,09 | 0,642-1 | 64 | 83 | ≤0,004 | 0,016 |
| GLSZM_SZHGLE | 0,788±0,1 | 0,585-0,99 | 83 | 59 | >76,3 | 0,033 |
| GLSZM_LZLGLE | 0,871±0,08 | 0,706-1 | 100 | 67 | ≤0,16 | 0,006 |
| GLSZM_LZHGLE | 0,833±0,08 | 0,664-1 | 77 | 83 | <36820 | 0,014 |
| GLSZM_NGLNU | 0,773±0,1 | 0,541-1 | 64 | 83 | ≤0,13 | 0,044 |
| GLSZM_ZP | 0,864±0,08 | 0,699-1 | 100 | 59 | >0,09 | 0,007 |
| GLSZM_GLV | 0,841±0,1 | 0,627-1 | 67 | 96 | >0,3 | 0,012 |
| GLSZM_ZSV | 0,864±0,08 | 0,690-1 | 64 | 83 | ≤393,4 | 0,007 |
